# Extracellular Vesicles in Corneal Fibrosis/Scarring

**DOI:** 10.3390/ijms23115921

**Published:** 2022-05-25

**Authors:** Vincent Yeung, Nikolay Boychev, Wissam Farhat, Dimitrios P. Ntentakis, Audrey E. K. Hutcheon, Amy E. Ross, Joseph B. Ciolino

**Affiliations:** 1 Department of Ophthalmology, Schepens Eye Research Institute of Mass Eye and Ear, Harvard Medical School, Boston, MA 02114, USA; nboychev@meei.harvard.edu (N.B.); wfarhat@meei.harvard.edu (W.F.); audrey_hutcheon@meei.harvard.edu (A.E.K.H.); amy_ross@meei.harvard.edu (A.E.R.); 2 Retina Service, Angiogenesis Laboratory, Department of Ophthalmology, Schepens Eye Research Institute of Mass Eye and Ear, Harvard Medical School, Boston, MA 02114, USA; dimitrios_ntentakis@meei.harvard.edu

**Keywords:** cell-cell communication, cornea, exosomes, extracellular vesicles (EV), fibrosis, microvesicles, scarring, therapeutic, wound healing

## Abstract

Communication between cells and the microenvironment is a complex, yet crucial, element in the development and progression of varied physiological and pathological processes. Accumulating evidence in different disease models highlights roles of extracellular vesicles (EVs), either in modulating cell signaling paracrine mechanism(s) or harnessing their therapeutic moiety. Of interest, the human cornea functions as a refractive and transparent barrier that protects the intraocular elements from the external environment. Corneal trauma at the ocular surface may lead to diminished corneal clarity and detrimental effects on visual acuity. The aberrant activation of corneal stromal cells, which leads to myofibroblast differentiation and a disorganized extracellular matrix is a central biological process that may result in corneal fibrosis/scarring. In recent years, understanding the pathological and therapeutic EV mechanism(s) of action in the context of corneal biology has been a topic of increasing interest. In this review, we describe the clinical relevance of corneal fibrosis/scarring and how corneal stromal cells contribute to wound repair and their generation of the stromal haze. Furthermore, we will delve into EV characterization, their subtypes, and the pathological and therapeutic roles they play in corneal scarring/fibrosis.

## 1. Introduction

The cornea is the anterior part of the human eye, and its optical transparency is vital for vision. The corneal epithelium establishes itself as the main barrier of protection against external insults [1,2]. The highly organized collagen extracellular matrix (ECM) that consists of collagen and distributed proteoglycans (PGs), and uniform spacing provides strength and transparency, thereby maintaining proper curvature of the cornea and producing an optical path that transmits light efficiently [3,4]. The corneal stroma has precise levels of elasticity and stiffness which are required to maintain the form of the corneal surface for consistent refractive power [5]. Throughout maturity, keratocytes are quiescent, showing neither mitotic nor apoptotic activity to any significant degree [6,7]. Corneal scarring/fibrosis represents an overactivated healing process of the corneal stroma. The persistent onset of corneal scars resulting from chemical exposure, mechanical trauma, or infectious keratitis can lead to the loss of corneal transparency and blindness [8,9].

In tissues, the trafficking of biological material across membranes is an evolutionarily conserved mechanism and is part of any normal cell homeostasis [10,11,12,13]. Such transport of materials is comprised of passive and active transport, including through microparticles and extracellular vesicle (EV) that can collectively maintain compartmentalization of important EV cargo [14,15]. In pathological states, aberrant activity of export machinery results in the misexpression of proteins, microRNAs (miRNAs), and other biomolecules [13,16,17,18]. The relevance of EVs in corneal scarring/fibrosis is gaining considerable traction in relation to physiological and pathological responses to corneal wound healing [12,19,20,21,22,23]. Currently, extensive research is being carried out to understand their role and mechanism(s) of action in the occurrence and development of corneal related diseases.

This succinct review will focus on the following aspects with regards to corneal scarring/fibrosis: clinical relevance and management, corneal stromal cell and EV functional roles, and novel therapeutic approaches.

## 2. What Is the Clinical Relevance of Corneal Scarring/Fibrosis?

Corneal trauma or injury results in regeneration or fibrosis dictated by several factors, including age, gender, medical and ocular health, and cause [24]. Corneal opacification is an anterior segment fibrotic eye disease, which may be acquired through trauma (injury, surgery, and chemical), infection (viral, bacterial, and fungal), inflammation (pterygia, and pinguecula), or acquired genetic disorders (inherited dystrophies) [25]. The term ‘corneal dystrophy’ specific to the genetic origin was first introduced in 1890 [26,27]. After cataracts, corneal diseases represent the fourth major cause of visual impairment and blindness worldwide, accounting for more than 2 million people [28]. The Vision Loss Expert Group of the Global Burden of Disease Study (2020) stated that corneal opacities accounted for 4.2 million people having moderate to severe visual impairment or blindness, while one study [29] previously stated corneal opacity represented 3.46% of global blindness, following cataract and glaucoma. The previously updated the International Classification of Corneal Dystrophies (IC3D), offering a complete worldwide peer-reviewed compendium on corneal dystrophy anatomic, clinical, histopathologic, and genetic classifications, including confocal images [30]. These classifications divided corneal dystrophies into four categories based on cellular origin, such as epithelial and subepithelial, epithelial-stromal transforming growth factor beta induced (TGFBI), stromal, and endothelial, ultimately recognizing over 20 subtypes. It is important to note that the inclusion of corneal ectasia diseases (keratoconus and pellucid marginal degeneration) into formal corneal dystrophy classifications remains contested, due to their variable origin.

The corneal wound healing and angiogenic responses are key properties in clinical settings, which reflect the eye’s ability to maintain homeostasis; the latter is dependent on ECM, vasculature, and the cellular responses to metabolic disease, infection, or inflammation [31]. Corneal regenerative repair and restoration involve a variety of matrix elements (fibronectin [FN1], collagen I and III, and tenascin C) and specialized cells (fibroblasts, fibrocytes, and myofibroblasts) for cross-linking; while keratocytes, Schwann and immune cells are the recognized resident cells that modulates a transparent stromal ECM [32]. To ensure successful recovery of the corneal epithelium and basement membrane zone (BMZ), different factors have been suggested for consideration: (1) the corresponding cells must heal first; (2) deep corneal injuries involving the posterior stroma have less capacity to heal than anterior ones; and, besides severity, (3) surface irregularity and genetic risk predisposition affect the ability to reinstate corneal transparency [33]. While these observations are taken into consideration, human corneas post-keratoplasty often recover between six months and a few years, where transparency is regained within two to three months [33]. Such considerations have been important in guiding clinical practice, particularly in cases of dense corneal scarring/fibrosis involving the healing process of the epithelium and stroma.

Acquired corneal trauma affects the corneal epithelial cells from which transforming growth factor β (TGFβ) from the injured epithelium enter the corneal stroma to trigger wound healing and stromal activation responses [34]. Corneal biomechanics are anisotropic and of considerate importance, as stromal rigidity is a dictating factor in the epithelial wound-healing response triggered by TGFβ [35]. Increased stromal stiffness after corneal injury has been attributed to the development of inflammation, corneal haze, and increase in myofibroblasts [36]. This is crucial information for corneal surgeons, since stromal rigidity can be manipulated by corneal cross-linking or keratorefractive procedures that promote apoptosis of myofibroblasts within the treatment area [37]. Furthermore, a healthy cornea absorbs strain in its anterior lamellae, but in case of disease, stress is assumed by its posterior layers [38]. The pathogenesis of corneal fibrosis/scarring is characterized by excess ECM production and corneal stromal activation is associated with excessive pro-inflammatory cytokines secretion [39]. However, it may also be the product of malfunctioning ECM degradation and turnover [40]. Thus, it is imperative that the ECM and BMZ reassembly outperforms the removal of damaged collagen fibers and fibrils to prevent extenuated fibrosis, which may result in corneal thinning and perforations [41].

An uninterrupted collagenous ECM is essential for the preservation of the cornea’s natural transparency and curvature. This ensures good vision by sustaining a clear central visual axis and functioning phototransduction. Corneal pathology (neovascularization, edema, and stromal fibrosis) can lead to diminished vision and severe cases would require corneal transplantation by keratoplasty. TGFβ is one of drivers of corneal scarring/fibrosis, and it is a common therapeutic target of interest for gene inhibition with respect to preventing and treating corneal fibrosis [39]. Interestingly, TGFβ secreted by myofibroblasts has been proposed to prevent nerve regeneration following corneal injuries from infections and photorefractive keratectomy [42]; causes donor corneas for keratoplasty to remain de-innervated [9]; and aids re-innervation of laser-assisted in situ keratomileusis (LASIK) flaps [43]. The density of corneal scarring following keratectomy is normally dependent on whether mitomycin C (MMC) was used, and could take at least one to two years for complete resolution [41]. A similar approach of photorefractive keratectomy combined with MMC may also be applied to stromal fibrosis and epithelial ingrowth frequently found in LASIK flaps [44]. Refractive surgeries have been categorized into healing patterns based on the severity of the subepithelial haze, where a faint haze usually heals within the first three months [45]. Persistent epithelial defects (PEDs) that do not heal within two to four weeks also lead to corneal scarring, due to the abnormal BMZ regeneration caused by missing laminins [33]. This is the common time frame for the development of alpha smooth muscle actin (αSMA) positive myofibroblasts [6]; therefore, it is imperative to heal PEDs by all acceptable measures such as lubrication, eyelid defect repair, epithelial debridement, tarsorrhaphy, cenegermin-bkbj, or amniotic membranes [46]. These measures may also be applied together with antimicrobials in cases of microbial ulcers of viral, bacterial, fungal, or rare acanthamoeba nature [47]. Regarding corneal cross-linking, fibrosis is the outcome of fibroblast or keratocyte activation, instead of myofibroblasts, and similar measures should be deployed if the epithelium has not healed by 10 days [2]. Collectively, all common types, causes and clinical features of corneal scarring/fibrosis in summarized in Table 1.

**Figure 1:**
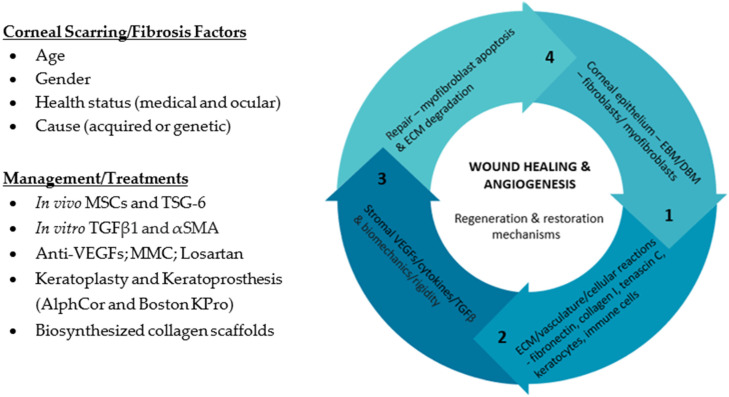
A schematic overview to corneal scarring/fibrosis: associated clinical factors; the corneal wound healing and angiogenesis response with the four major steps indicative of the regeneration and restoration mechanisms; and current management strategies.

Although effective treatments for corneal fibrosis/scarring currently exist, such as corneal transplantation and associated limbal stem cell transplantation, alternative therapeutic research approaches for corneal fibrosis/scarring models have been proposed. In vivo work [7] has shown the effectiveness of stromal mesenchymal stem cells (MSCs) in reducing αSMA expression [48], specifically through modulating secretion of tumour necrosis factor α (TNFα) and stimulated gene/protein 6 (TSG6) [49], while in vitro human culture models highlights the importance of the TGFβ1 signaling pathway in driving αSMA onset [50]. Anti-vascular endothelial growth factor (VEGF) treatments with bevacizumab and ranibizumab have been shown to prevent corneal vascularization [51] and enhance transplant graft survival [52]. Pharmaceutical options, such as topical MMC [53] and losartan [54], have been shown to aid in vivo corneal wound-healing response during surgeries prone to causing fibrosis, respectively. However, sufficient long-term clinical trials are lacking. Other efforts, such as the AlphaCor and Boston Keratoprosthesis [55], and biosynthesized collagen scaffolds [56], also hold promise by replacing the fibrotic tissue to restore normal corneal functioning, but true ECM replacement and replication of corneal tensile strength remain problematic [8]. These are important steps, since the clinical management of corneal scarring/fibrosis is based on corticosteroids and immunosuppressives (cyclophosphamide, azathioprine, mycophenolate, mofetil, and more), which are limited by their overall efficacy and long-term adverse risks [57]. This is also followed by surgical treatment options, such as keratectomy or keratoplasty, that require corneal transplants capped by donor cornea sources and significant graft rejection rates. Collectively, this has been summarized in Figure 1. Thus, new approaches are certainly needed, and identifying new insights behind corneal stromal cell biology will provide an in-depth understanding of corneal fibrosis/scarring formation.

## 3. How Does Corneal Stromal Cells Contribute to Corneal Fibrosis/Scarring?

The healthy cornea consists of 5 layers, namely, the epithelium, Bowman’s layer, stroma, Descemet’s membrane, and endothelium, all of which are essential for clear vision [4,58]. The corneal stroma is composed of tightly packed collagen fibrils intertwined with a matrix of PGs and glycoproteins (GPs) [34]. Due to their uniform spacing and parallel lattice-like arrangement, collagen fibrils are thought to give strength and promote corneal transparency [40]. The primary cell types of the cornea are epithelial cells, stromal keratocytes and endothelial cells; with epithelial cells being the most renewable and followed by keratocytes [7,34]. Additional cell types do exist in the cornea, such as dendritic bone marrow-derived immune cells, histiocytes, Schwann cells, and trigeminal nerve dendrites [59]. Corneal stroma injuries can cause changes in the phenotype of corneal stromal cells leading to corneal opacification and here we will delve into understanding how these changes occur.

### 3.1. Human Corneal Keratocytes (HCKs)

Human corneal keratocytes (HCKs) are a population of quiescent neural crest-derived mesenchymal cells residing between the collagen lamellae. Under normal physiological conditions, keratocytes display a stellate-like morphology and have a compact cell body [60]. The latter minimizes keratocyte surface area, and serves to reduce light scattering [61]. This is reinforced by the presence of aldehyde dehydrogenase class 1 (ALDH1) and transketolases (referred as crystalline proteins) that further reduce light scattering [5]. Collectively, stromal transparency is primarily dependent on these keratocyte’s features, as disruption in the quiescent keratocyte phenotype can lead to opacity.

Corneal keratocytes can secrete an array of ECM components, including collagen fibrils and PGs that remains vital for maintaining ECM metabolism and consequent transparency of the corneal stroma [9,34]. Multiple processes that include the synthesis and degradation of collagen molecules by matrix metalloproteinases (MMPs) contribute to maintaining this homeostasis [35]. In addition, PGs are important components of the ECM contributing to tissue organization in the corneal stroma, serving to regulate collagen fibrillogenesis, influencing keratocyte growth, modulating growth factor effects and maintaining stromal hydration. PGs consists of either a keratan sulfate, chondroitin sulfate, or dermatan sulfate as its core and other small leucine-rich proteoglycans (SLRPs), six of which are expressed in the corneal stroma: biglycan, decorin, fibromodulin, keratocan, lumican, and osteoglycin [62]. In particular, the keratan sulfate PG (KSPG) form consisting of lumican, keratocan, and mimecan has a role in regulating fibrillar spacing and fibril turnover. KSPGs plays a key role in establishing and maintaining corneal transparency by maintaining structural integrity of collagen fibrils.

Corneal keratocytes as mentioned are of mesenchymal origin, and, while normally quiescent, they maintain a phenotype that expresses cell-surface markers CD34 and CD133 [61,62]. They are characteristically reported to express keratocan more commonly, as well as the other SLRPs. At the intracellular level, the production of ALDH1 and transketolase as keratocytes is constant at the homeostatic level. However, disruption (via physical or chemical trauma) of the corneal epithelial barrier triggers stromal inflammation and induces morphological and phenotypic changes of keratocytes which are associated with their transformation to corneal fibroblasts [6,7,9,34].

### 3.2. Human Corneal Fibroblasts (HCFs)

Upon corneal stromal injury, a portion of keratocytes near the localized region of injury would either undergo cell death (apoptosis or necrosis), or a transformation process into a repair-phenotype of activated fibroblasts [8,36]. The resulting fibroblasts has a spindle-shaped morphology, possess multiple nucleoli, and lack cytoplasmic granules [39]. Fibroblasts are a proliferative and migratory cell type which secrete an array of repair-type ECM components, collagenases, and proteinases, that function in reconstructing the damaged stroma [63]. Not only do corneal fibroblasts serve to protect the stroma, but they act as sentinel cells to sense injury or infection by recognizing changes to the surrounding ECM, damage-associated molecular patterns (DAMPs) derived from damaged cells, pathogen-associated molecular patterns (PAMPs) derived from infectious microbes, and host cytokines through various receptors [64]. They play a role in recruiting inflammatory cells to the cornea during innate and acquired immune responses, contributing to the type of infiltrating cells through expressing adhesion molecules and chemokines [65,66].

The transition of corneal keratocytes to fibroblasts is accompanied by a marked decline in ALDH and keratocan expression [49]. Furthermore, the presence of corneal fibroblasts increases the secretion of components, such as FN1, cell-ECM adhesion molecules (integrins), MMPs (MMP-1, -3, and -9), collagen (type I and III), and PGs as phenotypic features differing from keratocytes [7,41,67]. Whilst the reverse can be reported with reduced levels of keratocan and keratan sulfate in corneal fibroblasts. Concomitantly, differences in their contractile phenotype related to wound contraction during healing is evident, with keratocytes being noncontractile [7,9,12].

### 3.3. Human Corneal Myofibroblasts (HCMs)

As the corneal healing process advances into the last stages, corneal fibroblasts can further differentiate into myofibroblasts under the synergistic action of serum and TGFβ1 [63]. Myofibroblasts are not prevalent in the uninjured cornea and are activated upon injury. Myofibroblasts are characterized by increased cell size and an ultrastructure akin to those of smooth muscle cells with expression of the contractile stress fiber, αSMA in the corneal stroma [6,9,20,33,39]. Although myofibroblasts contribute to the wound-healing process, excessive presence of myofibroblasts can give rise to corneal scarring/fibrosis by releasing an abundance of ECM that includes collagen, FN1, and PGs [8,9]. Collectively, this excretion may serve as an attempt to recapitulate developmental pathways, designed to regenerate functional tissue; additionally, it may also provide mechanical stability and protection against other pathologies such as infections (that could result in loss of the eye).

TGFβ1 plays a significant role in driving myofibroblast differentiation and their downstream effects are mediated by the (Mothers against decapentaplegic homolog (SMAD))-dependent or -independent pathways [50,68]. SMADs are intracellular proteins which transduce TGFβ1-dependent signals; more specifically, SMAD2/3 are phosphorylated, and then lead to nuclear translocation to regulate the transcription of ECM components [7,39,57]. Under sustained and unremitting activation of this pathway, these myofibroblasts express αSMA, but also desmin, vimentin, and integrins that are associated with focal adhesion involved in the assembly of FN1 fibrils [7,9,34,41,65,69]. These components are a multi-faceted apparatus that allows myofibroblasts to exert mechanical force and contribute to wound matrix organization and wound contraction. Myofibroblasts can secrete excessive levels of collagen (type I), FN1, hyaluronan, MMPs, and PGs [33,69,70]. Collectively, these molecules and pathways are interlinked, which governs ECM remodeling and mediates wound closure; whilst corneal transparency can be restored by understanding the balance between ECM synthesis and degradation as corneal integrity and transparency can be maintained.

## 4. What Are Extracellular Vesicles (EVs)?

**Figure 2:**
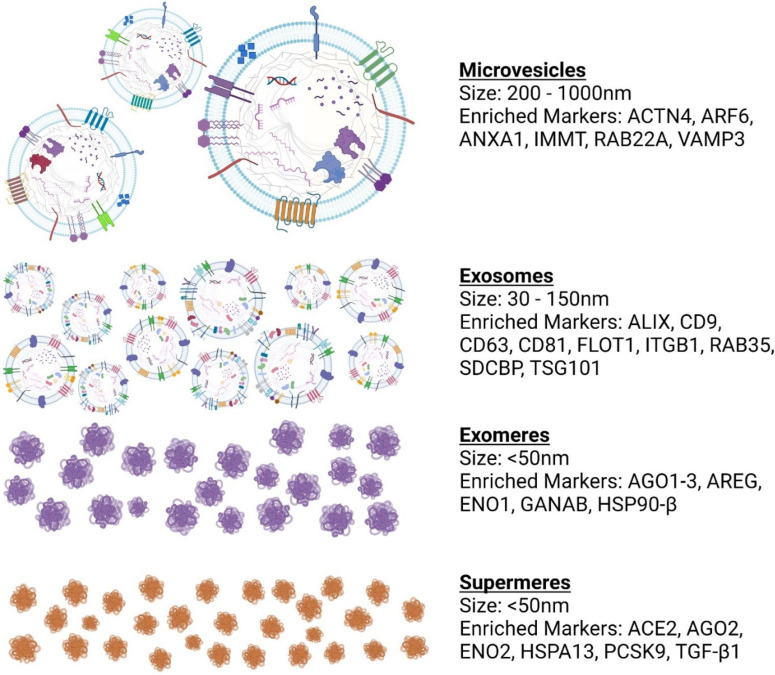
A schematic overview of different extracellular vesicles (EVs) types that include microvesicles, exosomes, exomeres, and supermeres. Microvesicles are typically formed by the outward budding of the plasma membrane. Exosomes are derived from multivesicular bodies (referred to as intraluminal vesicles) during formation and can be derived from the cell surface. The biogenesis of exomeres and supermeres remain unknown and are complexes of proteins and nucleic acids that are not membrane enclosed. Collectively, each of these EVs types are enriched in distinct markers that currently defines their composition.

EVs are a heterogeneous class of lipid bilayer-closed membranous structures comprising of microvesicles (MVs) and exosomes, which are shed from the plasma membrane or originate from the endosomal system, respectively [16]. Initially, EVs were described to jettison waste moieties and compounds, but recent research has focused on their capacity to exchange components between cells including proteins, lipids, and nucleic acids [71]. EVs are gradually known as one of the paracrine signaling mechanism(s) in cell homeostatic processes or as a repercussion of disease-influencing developments. Although the term EVs is now used to refer to all secreted membrane vesicles. EVs is the preferred term for all research investigations, and subtypes should be defined by physical and biochemical characteristics as reported by MISEV2018 [67]. To our knowledge of their biogenesis and secretion has pinpointed two EV subclasses: MVs and exosomes [15,18,72]; with the different EV types summarized in Figure 2.

The generation of exosomes and MVs have different modes of biogenesis with their formation occurring at distinct sites within the cell. Yet, both mechanisms share sizable overlap with membrane-trafficking processes and sorting machineries. The cargoes scheduled for secretion within EVs must be targeted to the site of production, either at the plasma membrane (for MVs) or at the limiting membrane of the multivesicular bodies (MVB) (for exosomes).

### 4.1. Microvesicles

MVs were initially described as subcellular material originating from platelets from plasma and serum [73], with later research showing the release of these vesicles in stimulated neutrophils [69]. Although initial work was predominantly focused in blood coagulation [70,74], more recently, extensive research has shown their role in cell-to-cell communication in various cell types, with this in cancer cells, where they are referred to as oncosomes [72]. They are generated by the outward budding and fission of the plasma membrane and the sequence release of vesicles into the extracellular space [15,75].

While blebbing from the plasma membranes has been recognized to form apoptotic bodies during apoptosis [76,77], there has been emerging mechanisms of how MVs are released from the plasma membrane during normal physiological conditions. MV biogenesis is a multifaceted approach that requires molecular rearrangements within the plasma membrane, including changes in protein and lipid components, and in Ca^2+^ levels [75]. The rearrangements in the asymmetry of membrane phospholipids (exposition of phosphatidylserine to the inner leaflet to the cell surface) can be attributed to Ca^2+^-dependent enzymes [77,78,79], which reforms the underlying actin cytoskeleton and physical bending of the membrane, which favors MVs formation and budding [75,80]. It has been reported that a genetic defect in the scramblase enzyme suppresses the exposure of phosphatidylserine on blood platelets and production of procoagulant-containing MVs [81]. Also, pharmacological depletion in cholesterol levels, which is abundant in MVs, has been shown to impair MV generation in activated neutrophils [82].

Lipids and other membrane-associated cargoes are bound to regions of MV budding, namely through their affinity for lipid rafts or oligomeric cytoplasmic proteins, by their anchoring to plasma membrane lipids [83,84,85]. Cytosolic components fated for secretion into MVs require their binding to the inner leaflet of the plasma membrane where this is dependent on plasma membranes anchors (myristoylation, palmitoylation, or prenylation) and the establishment of high-order complexes, which concentrates them to the small membrane domains which forms MVs that will bud [83,84,85,86,87]. It remains unclear how EV cargo such as nucleic acids are found in MVs, but the mechanism(s) of action of this process remains to be unraveled.

### 4.2. Exosomes

The term exosome, was initially used to describe membrane vesicles that were released by reticulocytes during differentiation that ranges from 30–150 nm in diameter [71]. In brief, exosomes are an EV subclass of endosomal origin appearing as intraluminal vesicles (ILVs), formed by the inward budding of endosomal membrane during maturation of MVBs which are intermediates within the endosomal system, and secreted upon fusion of the MVB with the plasma membrane. Early studies reported that exosomes were to be secreted by B lymphocytes [88] and dendritic cells [89] and were considered as moieties for anti-tumoural immunological responses. In the past decades, exosome secretion has been extended to numerous cell types, and its implication in intracellular communication is one of many paracrine mechanism(s) that drives normal homeostatic and pathological pathways; yet, exosome formation in different cell types is akin to one another [16,22,90,91,92,93].

The formation of MVBs and ILVs are processed in part, by the endosomal sorting complex required for transport (ESCRT) complex (containing 30~ proteins). The ESCRT machinery has four distinct ESCRT complexes (ESCRT-0, -I, -II, and -III) that acts in a stepwise manner. The ESCRT machinery and the presence of other proteins, such as programmed cell death 6-interacting protein (ALIX), vacuolar protein sorting associated protein 4 (VPS4), and vacuolar protein sorting-associated protein (VTA1), revealed various roles for ILV biogenesis. Their inactivation affects either the secretion or the composition of the secreted vesicles, indicating that these ESCRT components could act selectively on MVB and ILV subpopulations fated for secretion as exosomes [18,94,95].

Exosomes can also be formed in an ESCRT-independent manner. It was first reported ceramide generation by neutral type II sphingomyelinase was key for exosome biogenesis [96]. Furthermore, proteins of the tetraspanin family (CD9, CD63, CD81, and CD82) has been shown to regulate ESCRT-independent endosomal sorting of different cargoes to exosomes [97,98,99,100]. Moreover, tetraspanins also can regulate the intracellular routing of cargoes such as integrins [94,95], towards MVBs, which indicates that impairment may affect the generation of exosomes. Thus, it seems that both ESCRT-dependent and ESCRT-independent mechanisms govern exosome biogenesis, and their contribution may vary depending on their cargo and the cell type.

As mentioned above, sorting of MVBs is largely dependent on endosomal sorting machineries and trafficking are governed by small Ras-associated (RAB) GTPase proteins that are essential for regulating transport between different endosomal compartments [98,101,102]. Following this, the final step of exosome release involves fusion of MVBs with the plasma membrane to release ILVs as exosomes, a process probably mediated from the soluble N-ethylmaleimide-sensitive fusion attachment protein receptor (SNARE) protein family [103]. In brief, members of this family are categorized as vesicular SNAREs (v-SNARE) located on the vesicle’s membrane and target SNAREs (t-SNARE) located on the membrane of acceptor compartments [15,104]. This process is proposed to allow the SNARE proteins to form complexes between the MVB and plasma membrane to mediate fusion; thus, allowing the release of ILVs, termed as exosomes, often represented by a heterogeneous population that differs in their molecular composition.

**Figure 3:**
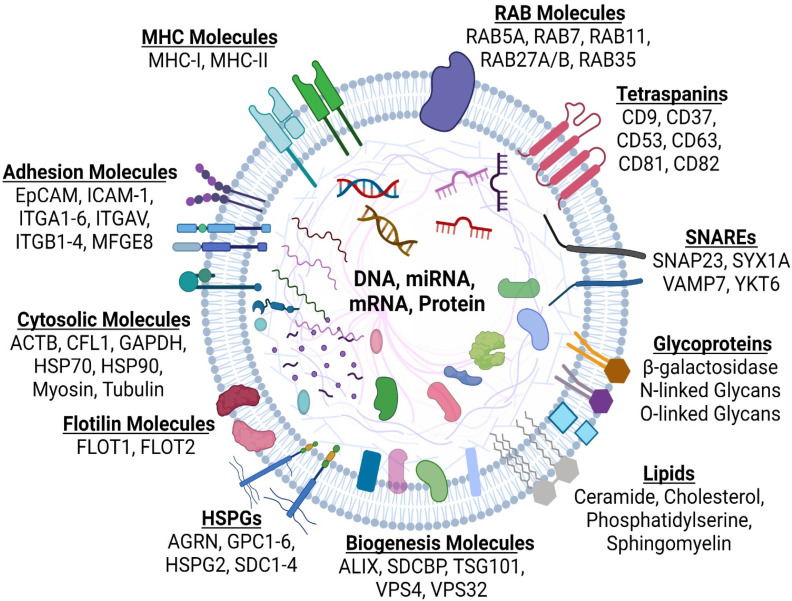
Typical structure and molecular composition of exosomes. Exosomes are surrounded by a phospholipid bilayer enriched in lipids such as ceramide, cholesterol, phosphatidylserine, and sphingomyelin. They are enriched in glycoproteins such as β-galactosidase, and N-linked or O-linked Glycans. Exosomes are enriched in proteins associated with biogenesis, such as programmed cell death 6-interacting protein (ALIX), syntenin-1 (SDCBP), tumor susceptibility gene 101 (TSG101), and vacuolar protein sorting 4 and -32 (VPS4 and VPS32). Upon trafficking of multi-vesicular bodies encompassing exosomes, they express small GTPase Ras-related proteins 5A, -7, -11, -27A/B and -35 (RAB5A, RAB7, RAB11, RAB27A/B, and RAB35). When MVBs fuse with the plasma membrane to release exosomes into the extracellular space, they also express soluble *N*-ethylmaleimide-sensitive fusion attachment protein receptor (SNARE) proteins such as synaptosome associated protein 23 (SNAP23), syntaxin1a (SYX1A), vesicle associated membrane protein 7 (VAMP7), and YKT6 V-SNARE Homolog (YKT6). Exosomes are enriched in; tetraspanin proteins, such as CD9, CD37, CD53, CD63, CD81, and CD82; flotillin (FLOT) molecules, such as FLOT1 and FLOT2; major histocompatibility complex-I and -II (MHC-I and -II); adhesion molecules for example epithelial cellular adhesion molecule (EpCAM), intercellular adhesion molecule-1 (ICAM-1), integrin subunit α1-6 (ITGA1-6), integrin subunit αV (ITGAV), integrin subunit β1-4 (ITGB1-4), and lactadherin (MFGE8); heparan sulfate proteoglycans (HSPGs) that include agrin (AGRN), glypican 1-6 (GPC1-6), perlecan (HSPG2), and syndecan1-4 (SDC1-4). Exosomes can also contain cytosolic proteins that include actin (ACTB), cofilin1 (CFL1), glyceraldehyde 3-phosphate dehydrogenase (GAPDH), heat shock protein 70 and -90 (HSP70 and HSP90), myosin, and tubulin. The exosomal surface molecular and internal cargo serves to mediate intracellular communication between different cell types within the body, thus functioning differently in either normal homeostasis or pathological conditions.

In brief, the molecular composition of exosomes is summarized in Figure 3, and the list of other molecules consisting of lipids, proteins, DNA, mRNA, and non-coding RNAs has been characterized by the development of databases such as ExoCarta [105], Vesiclepedia [106], EVpedia [107], and exoRBase [108]. The nature and abundance of exosome cargo are often influenced by the physiological or pathological state of the donor cells with the stimuli modulating the molecular mechanism(s) that govern their pro duction and release. As reported for exosomes, the expression of the major histocompatibility complex (MHC) class II [104] promotes MVB formation and subsequence exosome release, probably by recruiting sorting machineries that will promote MVB and ILV generation. These observations extend to exosomal membrane cargoes that reach MVBs from the Golgi apparatus or internalized from the plasma membrane before being sorted to ILVs during endosome maturation [14,109]. In this context, the proteins syntenin [101], syndecan [102], ADP-ribosylation factor 6 (ARF6) [110,111], β1 integrin receptors [112,113], MHC class I molecules [114,115], and membrane type 1-matrix metalloproteinase 1 (MT1-MMP, also known as MMP14) [116,117], seem to be potential regulators of the crosstalk between endocytic recycling and endosomal targeting of loading cargo proteins onto exosomes.

### 4.3. Exomeres and Supermeres

More recently, further refinement in EV isolation methodologies (via asymmetric flow field–flow fractionation) has led to the identification of a new type of small (<50 nm) non-membranous extracellular nanoparticle, termed “exomeres” [118,119]. These small nanoparticles lack an encompassing membrane but contains a unique repertoire of bioactive components such as protein, lipid, DNA and RNA profiles, and N-glycosylation. Enrichment of proteins controlling glycan-mediated protein folding control (CALR), glycan processing (GANAB, HEXB, and MAN2A1), heat shock protein 90 AB1 (HSP90AB1), enolase 1 (ENO1), gelsolin (GSN), and phosphoglycerate kinase 1 (PGK1) were all reported to be enriched in exomeres. Together, this suggests a distinct biological function, although their biogenesis, function, and distribution remain poorly understood.

More recently, “Supermeres” (supernatant of exomeres) have been reported as novel distinct nanoparticles [120]. Supermeres were described to be morphologically and structurally distinct from exomeres as determined by fluid-phase atomic force microscopy. Interestingly, they exhibited a different cellular uptake kinetic than small EVs and exomeres in vitro and a greater uptake in vivo. A thorough analysis of the supermere component shows protein enrichment in different diseases ranging from the amyloid precursor protein (APP) and amyloid beta precursor like protein 2 (APLP2) in Alzheimer’s disease; angiotensin I converting enzyme (ACE), ACE2, and proprotein convertase subtilisin/kexin type 9 (PCSK9) in both cardiovascular and COVID-19; argonaute-2 (AGO2), cellular–mesenchymal–epithelial transition factor (MET), and TGFBI in cancers [120]. RNA sequencing analysis highlights the presence of numerous RNA species, and it is the identification of this array of bioactive moieties with distinct uptake kinetics that makes these nanoparticles highly promising as novel biomarkers for disease. It remains to be seen as this new classification has not been translated into the larger EV class of nanoparticles and research is required to address the functional and proteomic comparisons as part of deciphering the mechanism(s) as described [121] by which EVs play in paracrine signaling pathways.

## 5. What Roles Do Extracellular Vesicles Play in Corneal Fibrosis/Scarring?

In the cornea, bidirectional communication between the corneal epithelium and stroma plays a critical role in corneal wound repair. The aberrant activation of multiple processes that includes cell death, migration, proliferation; myofibroblast differentiation; and ECM remodeling can lead to corneal fibrosis that is accompanied with inflammation and neovascularization [8,12,31,33,36,54]. It is increasingly understood that cell-to-cell communication by paracrine mechanism(s), precisely by EVs have shown to influence physiological and pathological responses in relation to corneal wound healing.

One of the first papers studying EVs in the cornea identified an abundant expression of FN1 following keratectomy injury, but this early study also described the appearance of membrane-bound particles within the anterior stroma 3 days following a keratectomy [122]. Also, following injury of the corneal epithelium, the presence of EVs has been detected following basement membrane reformation. Similarly, the detection of vesicle-like structures at the basal edge of epithelial cells following injury in a different model has been shown, as the migrating epithelium deposits a new basement membrane [123]. These structures are akin to MVs that resulted from outward budding and perhaps provides an indication that EVs are involved in governing communication within the cornea.

Following these early studies, further investigations have examined the potential roles of corneal epithelial-EVs in corneal wound healing. The presence of EVs in the corneal stroma and their fusion with corneal keratocytes in vitro induce myofibroblast differentiation has been documented following anterior stromal keratectomy [12]. Furthermore, these findings extended to show epithelial-EVs induced vascular endothelial cell proliferation and ex vivo aortic ring sprouting. More recently, our laboratory found that epithelial-EVs released by corneal epithelial cells encapsulate provision matrix proteins that triggers corneal fibroblast to myofibroblast differentiation, while also contributing to elevated contractility, proliferation, and migration [20]. In a similar study, it was shown corneal epithelial-EVs have the capacity to influence the transdifferentiation of human conjunctival and corneal epithelial cells [124], or EVs pre-treated with thrombospondin-1 promoted tissue remodeling and repair [125]. More recently, our study examined the paracrine crosstalk from the corneal stromal (keratocytes, fibroblasts, and myofibroblasts) EVs and how they influence corneal epithelial wound healing [23]. The study provides evidence that corneal myofibroblast EVs compared to keratocyte- or fibroblasts-EVs contain distinct cargo proteins that promote corneal epithelial cell proliferation, migration, and motility. This provides evidence that EV-mediated communication between the epithelial and stroma influences corneal wound healing and development of corneal scars.

Currently, stromal cells (adipocytes, fibrocytes, fibroblasts, and MSCs), and their secreted EVs have been proposed to orchestrate pathological changes in models of cardiovascular diseases [126] and oncology [17,97,98,102,127]. In particular, when examining the therapeutic action of MSC-EVs, it has been reported to show restorative functions in different organs in various disease models [93,128,129,130,131,132,133]. This original promise of MSCs would permit the development of therapies where donor cells would participate in wide reparation and regeneration of the injured or diseased tissue. Indeed, over time subsequent studies clarified that the improvement and restoration in various disease models could not be accounted for by donor cell engraftment; instead, paracrine factors play a major, if not the sole, role in the mechanisms of MSC therapeutic action [22,127,129,130,133,134,135,136].

With an emphasis on the cornea, it has been reported that administration of MSC-EV can accelerate corneal epithelial wound healing [19] and reduce pro-inflammatory cytokines level in a mouse in vivo model of desiccating stress [137]. This was highlighted more recently when reporting that umbilical cord MSC-EVs in combination with an autophagy activator significantly enhanced corneal epithelial cell function, alleviated corneal defects, and reduced levels of apoptotic markers in vivo [138]. Furthermore, these MSC-EV observations extends to influencing corneal stromal cells by promoting ECM synthesis and changing collagen and MMP levels [139]. Also, human saliva-EVs has also been reported to regulate stromal cell migration and wound healing [140]. Overall, these studies have been summarized in Table 2 and provide evidence that EVs may serve a novel application as therapeutic carriers of cargo that could promote scarless corneal healing.

## 6. Can We Utilize Extracellular Vesicles in Treatments Regiments in Corneal Fibrosis/Scarring?

Over the past decade, different approaches in treating ocular-related diseases by gene silencing [141,142,143], drugs [144,145,146], and oxygenating technologies targeting particularly the wound-healing mechanism [147,148] have been explored. There has been some success, yet further research is warranted for novel treatments and cell-free EV based therapy is emerging as a promising approach because of its advantages over ongoing options in many diseases [135,139,149,150]. There has been a consensus that EVs are natural paracrine mediators of most existing cells, holding a vital role in cell-to-cell communication [151]. Understanding the bioactive cargo of EVs and their mechanism(s) of action in different disease models will certainly enhance our newfound knowledge that can be applied translationally. Different therapeutic approaches have been touted by loading EVs with miRNA, proteins, or drugs for improving precision-medicine efforts to treating different pathologies [152]. As discussed before, MSC-EVs could hold great promise as they are gradually recognized as effective drug delivery systems that can overcome earlier drug-stability and immunological issues [136]. This is of importance in corneal scarring/fibrosis, where the homeostatic nature of wound healing entailing of inflammation, proliferation and regeneration is disrupted [153]; meanwhile, MSC-EVs could induce a potent wound-healing process under the hypoxic and inflammatory conditions that typically characterize chronic injury [154].

Clinical trials have confirmed the efficacy and safety of MSCs in wound treatment [154], but their technical utility is limited [155]. The literature on pre-clinical in vivo and in vitro studies evaluating MSC-EVs in promoting wound healing has been reported. Umbilical cord MSC-EV enriched in specific miRNAs (miR-21, -23a, -125b, and -145) suppressed the onset of myofibroblasts [156]. It was reported that specifically inhibiting those miRNAs were essential for the myofibroblast-suppressing and anti-scarring functions of umbilical cord MSC-EVs. Furthermore, EVs derived from adipose tissue-derived stem cells were shown to be taken up by fibroblasts to promote cell proliferation and migration in vitro, whilst accelerating wound healing in a full thickness incision wound mouse model [157]. Collectively, these reports have shown reduced scar formation and myofibroblast accumulation [149,156,157].

In addition, numerous studies also showed adipose-derived MSC-EVs suppressed TGFβ/SMAD fibroblast signaling and apoptosis with MSC-EV treatment. Adipose-derived MSC-EVs were shown to promote full thickness skin wound repair in vivo [150]. In a similar study, the application of adipose-derived MSC-EVs decreased the expression of αSMA, collagen I and III, p-Smad2/pSmad3 on a hypertrophic scar in mice with full-thickness defects created on the dorsal skin, whilst demonstrated accelerated wound healing and less collagen deposition [158]. Specifically, umbilical cord MSC-EV enriched in specific miR-21, -23a, -125b, and -145 suppressed the onset of myofibroblasts (αSMA onset and collagen deposition) by inhibiting TGF-β2/SMAD2 pathways [156]. It is suggested these studies provide evidence that MSC-EVs suppresses TGFβ/SMAD fibroblast signaling.

Other findings have demonstrated that MSC-EV application can also increase re-epithelialization (by promoting proliferation and viability) and ECM remodeling. It was shown that adipose derived MSC-EVs administration significantly accelerated wound closure and re-epithelization in an established in vivo 7mm full-thickness cutaneous wound mouse model [159]. In part, the therapeutic functional capacity of MSC-EVs could be due to the upregulation of proliferative markers (cyclin D1 and D2), growth factors (EGF, FGF2, and VEGFA), and activation of AKT and ERK signaling pathways in fibroblasts, keratinocytes, and endothelial cells. Similarly, MSC-EVs loaded with miR-27b improved cutaneous wound healing in vivo and improved proliferation and migration of keratinocytes and skin fibroblasts in vitro [160]. The therapeutic capacity of MSC-EV demonstrated that it promoted proliferation and angiopoeisis in endothelial progenitor cells in vitro [161]. Furthermore, it was reported that MSC-EVs significantly reduced the ulcerated wounded area in a diabetic foot ulcer in vivo rat model. Similarly, MSC-EVs exhibited a therapeutic response in facilitating the wound regeneration by promoting the formation of blood vessels in a streptozotocin (STZ)-induced diabetic rat model [162]. Interestingly, it was demonstrated that both adipose tissue or bone-marrow derived MSC-EVs promoted wound-healing effects of diabetic wounds in vivo; although the cargo from bone marrow MSC-EVs were preferentially involved in triggering cellular proliferation, whereas adipose MSC-EVs were correlated with pro-angiogenic responses [163]. Together, these studies suggest that MSC-EVs can contribute to re-epithelization and wound healing by promoting proliferation and viability of fibroblasts, keratocytes, and endothelial cells.

It has been proposed that chronic wounds can be characterized by an abnormal inflammatory state and potentially mitigated by the immunomodulatory capacity of MSC-EVs. Evidence of MSC-EVs modulating the balance of macrophages due to their upregulation of anti-inflammatory cytokines and the “M2”-like phenotype has been shown [164]. The application of MSC-EVs was shown to enhance cutaneous wound healing in a STZ-induced diabetic rat model, and, in part, the TLR4/NF-κB/STAT3/AKT regulatory signaling pathway that plays a role in regulation of macrophage plasticity. Similarly, MSC-EVs labelled with iron oxide was readily found to promote human umbilical vein endothelial cell proliferation, migration, and angiogenesis both in vitro and in vivo, but, importantly, reduced scar formation and collagen expression in a skin thickness burn rat model [165]. Similarly, bone marrow MSC-EVs was shown to promote cutaneous wound healing by triggering the macrophage phenotype to an immunomodulatory “M2” phenotype in vitro. Also, the proliferative and migratory functions of fibroblasts were elevated, whilst not fully triggering myofibroblast onset [149]. There is evidence that MSC-EVs regulates the inflammatory microenvironment that may enhance the response to injury and reduce scarring/fibrosis. While these wound-healing studies were not conducted in corneal models, their findings suggest that MSC-EVs may have the potential to enhance the corneal response to injury and reduce scarring/fibrosis.

With the ever-growing literature on EVs in various disease models, studies related to the cornea are beginning to grow. The earliest evidence of EV involvement in corneal wound healing began in 1987 from in vivo rabbit keratectomy research [122]. The functional role of cell-to-cell communication has shown to be mediated by EVs during corneal wound healing, where the corneal epithelium interacts with stromal keratocytes, fibroblasts, and vascular endothelial cells [11,12,20,21], while corneal stromal EVs interact with corneal epithelial cells [23]. In another study, limbal stromal cell derived exosomes have been reported to promote proliferation and regeneration of limbal epithelial cells [13].

Approaches to load MSC-EVs with therapeutic moieties, such as miRNA, have been reported to reduce corneal scarring/fibrosis, inflammation, and lead to a scarless recovery [166]. This study noted the ability of corneal stromal EVs delivering miRNAs in vivo to mimic the regenerative attributes of corneal stem cells two weeks following injury by neutrophil reduction. Similarly, other ocular-based research has demonstrated that EV-associated adeno-associated virus type 2 (EV-AAV-2) from 293T cells demonstrated a deeper penetration in the retina via an intravitreal injection, suggesting an effective method for intravitreal gene transfer [13]. Also, one study reported that MSC-EVs over-expressing miR-126 successfully suppressed the HMGB1 signaling pathway and suppressed hyperglycemia-induced retinal inflammation in vivo [167]. So far, there has been limited research on loading drugs or moieties onto EVs to treat ocular-related diseases or injuries, but these published studies could provide an alternative insight that could treat corneal scarring/fibrosis.

EV/exosome-based therapies for corneal disease have been suggested to be advantageous to current leading treatments, such as transplantation or stem cells due to availability, accessibility, safety, regulatory affairs, and cost [168]. Further benefits may include topical administration, storage at room temperature, and withheld potency post-lyophilization [169,170,171,172]. However, notable challenges also exist, such as the need of scalability and good manufacturing practices [168]. Although the therapeutic application of EVs and exosomes in promoting wound healing and regeneration during corneal scarring/fibrosis is gaining traction, clinical trials confirming the specific functional mechanisms of these very promising avenues would be needed for integration into real-world clinical management.

## 7. Summary Statement

As the EV-research field continues to mature, the evidence for their important role in modulating the corneal microenvironment is compelling. Epithelial and stromal cell derived EVs clearly exert multiple, and highly complex effects that appear to mediate various outcomes, such as myofibroblast differentiation, epithelial cell migration and proliferation, ECM re-structuring (matrix contraction and reorganization), and epithelial-stromal interactions (basement membrane dissolution and reformation). It is likely that different EV subsets have varied effects depending on the cell origin, relative abundance, target cell and, now, the emerging novel EV subtypes (exomeres and supermeres). Decoding the EV roles in the cornea will require meticulous isolation and biochemical analyses to define the reproducible surface markers consistent with EV subsets and their associated molecular properties. By understanding the EV’s functional cargo and their influence on wound healing and corneal scarring/fibrosis may help in understanding healing/development mechanisms that could determine clinical outcome.

Over the past decade, more than >800 MSC therapy clinical trials have been registered and their immunomodulatory and regenerative properties have made MSCs one of the most lucrative pursued cellular therapies. Currently, the only stem cell treatments approved by the Food and Drug Administration (FDA) are products used to treat certain cancers and disorders of the immune and blood system [146]. In the cornea, there is increasing preclinical evidence to show that cell replacement therapies, such as limbal, oral mucosal, or pluripotent cells can restore the corneal epithelium. The advantages of using MSC therapies in tissue repair, i.e., their relatively wide differentiation capacity, immunoregulatory feature, tolerable safety profile, and high paracrine ability, including EV release, make these cells an important material for investigation. Although, growing evidence suggests the MSC paracrine properties via EVs have antifibrotic and immunomodulatory features. Preclinical studies report the therapeutic properties of topical application MSC-EVs in promoting corneal wound healing, but their application in human studies is scarce as the FDA have not approved any exosome/EV based treatments. Additional EV research is required to understand the challenges MSC-therapies are still facing such as the heterogeneity of preparation, source of cells, culture conditions, expansion, dose and delivery route. Understanding these challenges are essential for bringing a cell therapy to the clinic and by working closely with the FDA, this ensures the best chance that an Investigational New Drug (IND) application has success in a clinical trial. Collectively, there remains many challenges, yet the exciting advancements in ocular research and potential prospect of utilizing EVs may serve as an alternative approach for corneal-related treatments.

## Figures and Tables

**Table 1:** Common types and cause of corneal scarring/fibrosis diseases with corresponding clinical signs, symptoms, duration, and management options.

Type	Cause	Signs	Symptoms	Duration	Management
Epithelial (Basement Membrane)	Degenerative; trauma	Abnormal basal epithelial cell adhesion	Asymptomatic; Pain; Vision Impairment; Monocular Diplopia (Ghost Images)	Fluctuates	Saline; Ointment; Antibiotic/Antifungal
Endothelial (Fuchs)	Mostly without known inheritance. Proposed to have autosomal dominant mutations	Guttae; Edema; Pigment dusting; Bullous keratopathy; Vascularization	Fluctuating vision with progressive loss; Pain; Photophobia; Epiphora; Corneal haze and curvature change	Progressive	Saline; Ointment; Contact lenses; No Cure
Trauma	Accidents; Injuries; Burns	Cornea rupture; Endophthalmitis; Hyphaemas; Ulcers	Pain; Vision impairment may result in blindness	Fluctuates	Antibiotic/Antifungal; Corneal transplant and keratoplasty
Drugs/Infection (Stevens–Johnson Syndrome)	Drugs (NSAIDs; sulphonamides); Infection (HSV)	Bullous; Epidermal necrolysis	Vision impairment may result in blindness	Fluctuates; Progressive	Cease drug source; Immunosuppression; Corneal transplant and keratoplasty
Infection (Trachoma)	Bacterial (Chlamydia)	Entropion; Trichiasis; Vascularization	Vision impairment may result in blindness	Fluctuates; Progressive	Antibiotics; Corneal transplant and keratoplasty
Infection (Leprosy)	Bacteria	Cataract; Keratitis; Ulcers; Uveitis; Vascularization	Vision impairment may result in blindness	Progressive	Combination drug therapy

**Table 2:** Studies highlighting the roles of EVs in corneal wound-healing models.

References	Key Study Findings	Biological Model	Vesicle Source	Vesicle Methods
Han et al., 2017 [12]	Human/mouse corneal epithelial EVs induced endothelial cell proliferation and ex vivo aortic ring sprouting.	In vitro: corneal wound healing /neovascularization.	Human/mouse corneal epithelial cell line.	Total exosome isolation reagent + differential ultra- centrifugation
Samaeekia et al., 2018 [19]	Human corneal MSC-EVs can accelerate epithelial cell migration and proliferation in vitro and wound healing in vivo.	In vitro: corneal epithelial wound healing. In vivo: corneal debridement mouse model.	Human corneal MSCs derived from human cadaver corneas.	Differential ultra- centrifugation
McKay et al., 2020 [20]	Human corneal epithelial-EVs triggers corneal fibroblast to myofibroblast differentiation.	In vitro: interplay between epithelial EVs and corneal stroma.	Human corneal epithelial cell line.	Total exosome isolation reagent + differential ultracentrifugation
Lai et al., 2021 [125]	Human corneal epithelial-EVs treated with thrombospondin-1 (TSP-1) protected hypoxia-induced paraptosis in epithelial cells and promoted wound healing.	In vitro: interplay between corneal epithelial EVs (with TSP-1) and hypoxia-induced epithelial cells.	Human corneal epithelial cell line.	Differential ultracentrifugation
Yeung et al., 2022 [23]	Human corneal myofibroblast EVs promote epithelial cell migration, proliferation, and motility.	In vitro: interplay with corneal stromal EVs and epithelial cells.	Human primary corneal fibroblast /myofibroblast.	Differential ultracentrifugation
Ramos et al., 2022 [124]	Human corneal epithelial EVs influences transdifferentiation of human conjunctival/epithelial cells	In vitro: interplay with epithelial EVs and conjunctival/epithelial cell.	Human corneal epithelial cell line.	Differential ultracentrifugation
Ma et al., 2022 [138]	Umbilical cord MSC-EVs in combination with an autophagy activator alleviated corneal epithelial defects and stromal opacity in vivo.	In vitro: interplay of MSC-EVs on epithelial cells. In vivo: corneal debridement mouse model.	Human primary umbilical cord derived MSCs.	Differential ultracentrifugation
Escandon et al., 2022 [140]	Human salivary EVs modulated human corneal stromal cell migration and wound healing.	In vitro: interplay of salivary EVs on primary corneal stromal cells.	Human saliva from health donors.	Human biofluids characterized by ExoView^®^ R100 platform

## Data Availability

Not applicable.
